# Comparative renal effects of angiotensin receptor neprilysin inhibitors and ACEi/ARB: a systematic review and meta-analysis

**DOI:** 10.1093/ckj/sfaf224

**Published:** 2025-07-11

**Authors:** Adrian Covic, Luminita Voroneanu, Anca-Elena Stefan, Crischentian Brinza, Alexandra Covic, Mehmet Kanbay, Viorel Scripcariu, Stefan Iliescu, Alexandru Burlacu

**Affiliations:** University of Medicine and Pharmacy “Grigore T. Popa”, Iasi; Hospital “Dr C.I. Parhon” – Department of Nephrology and Renal Transplant, Iasi; University of Medicine and Pharmacy “Grigore T. Popa”, Iasi; Hospital “Dr C.I. Parhon” – Department of Nephrology and Renal Transplant, Iasi; University of Medicine and Pharmacy “Grigore T. Popa”, Iasi; Hospital “Dr C.I. Parhon” – Department of Nephrology and Renal Transplant, Iasi; University of Medicine and Pharmacy “Grigore T. Popa”, Iasi; Institute of Cardiovascular Diseases “Prof. Dr George I.M. Georgescu” – Department of Cardiology; University of Medicine and Pharmacy “Grigore T. Popa”, Iasi; Institute of Cardiovascular Diseases “Prof. Dr George I.M. Georgescu” – Department of Cardiology; Department of Nephrology, Koc University School of Medicine, Istanbul, Turkey; University of Medicine and Pharmacy “Grigore T. Popa”, Iasi; Regional Institute of Oncology – Department of Surgery; University of Medicine and Pharmacy “Grigore T. Popa”, Iasi; University of Medicine and Pharmacy “Grigore T. Popa”, Iasi; Institute of Cardiovascular Diseases “Prof. Dr George I.M. Georgescu” – Department of Cardiology

**Keywords:** ARNI RASI, ARNI renal, neprilysin inhibitor, sacubitril valsartan ACE, sacubitril valsartan renin–angiotensin–aldosterone

## Abstract

**Background:**

Classical renin–angiotensin system inhibitors (RASI), such as angiotensin-converting enzyme inhibitors (ACEi) and angiotensin receptor blockers (ARB), have long been the foundation of treatment for patients with cardiovascular disease (CVD) and chronic kidney disease (CKD). The development of angiotensin receptor neprilysin inhibitors (ARNI) has introduced a valuable therapeutic option for patients with heart failure with reduced ejection fraction (HFrEF), reducing the risk of major cardiovascular events and becoming an essential component of treatment for this population. However, their effects on renal outcomes remain uncertain.

**Methods:**

We conducted a systematic review and meta-analysis to compare the renal effects of ARNI and RASI. Relevant studies were searched in the following databases from inception to 30 December 2024: MEDLINE (PubMed), Embase and Scopus. The primary outcomes assessed were: a ≥50% reduction in estimated glomerular filtration rate (eGFR) or progression to end-stage renal disease (ESRD), a composite measure of worsening renal function (serum creatinine increase of ≥0.5 mg/dL from baseline and a 25% decline in eGFR) and renal impairment (an increase of at least 0.3 mg/dL in creatinine levels). Additionally, a subgroup analysis of renal impairment in patients with HFrEF was performed. Secondary outcomes included hyperkalemia.

**Results:**

Our results suggested a 31% reduction in renal impairment with ARNI treatment compared with RASI and a 37% reduction in the odds of ≥50% decline in eGFR or ESRD. However, the pooled analysis for worsening renal function and hyperkalemia showed no apparent difference between ARNI and RASI. A subgroup analysis on a population with a reduced ejection fraction suggested a 37% lower odds of renal impairment with ARNI when compared with RASI. This study represents the largest and first systematic review and meta-analysis with clearly defined renal outcomes.

**Conclusion:**

Given that ARNI has been explored for indications beyond heart failure, further randomized controlled trials are needed to understand its renal effects better. Future research should determine whether ARNI provides a benefit in a purely CKD population or in a cardio-renal population, given that CVD is the leading cause of mortality in CKD patients.

## Graphical Abstract

**Graphical Abstract:**
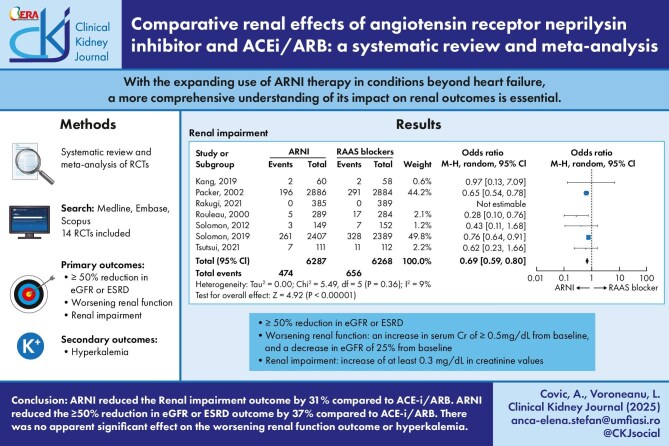

KEY LEARNING POINTS

- A 37% reduction in the odds of ≥50% decline in estimated glomerular filtration rate (eGFR) or end-stage renal disease with angiotensin receptor neprilysin inhibitors (ARNI) treatment compared with renin–angiotensin system inhibitors (RASI) [odds ratio (OR) = 0.69, 95% confidence interval (CI) 0.49–0.80, *P* < .0002].
- A 31% reduction in the odds of renal impairment with ARNI compared with RASI (OR = 0.69, 95% CI 0.59–0.80, *P* < .00001).
- There was no clear evidence of a difference between ARNI and RASI in terms of worsening renal function and hyperkalemia.

**What was known:**

- ARNI protects against renal impairment, ARNI users have a lower risk of lower eGFR or renal dysfunction.

**This study adds:**

- A clear view of the renal effects of ARNI compared with RASI, with well-defined renal outcomes based on the existing data.

**Potential impact:**

- A better understanding of the renal effects of ARNI when compared with classical renin–angiotensin system inhibition.

## INTRODUCTION

Chronic kidney disease (CKD) is a progressive condition that affects more than 10% of the general population worldwide, and studies predict that it may become the fifth cause of death worldwide by 2024 [1]. Major risk factors for CKD include arterial hypertension, diabetes mellitus and cardiovascular disease (CVD). Independently, recurrent episodes of acute kidney injury are a significant risk factor for CKD [2].

Cardiovascular disease (CVD) is the leading cause of morbidity and mortality in CKD. The relationship between CKD and CVD is bidirectional; CKD increases the risk for CVD, while CVD increases the risk of CKD progression [3]. The mechanisms involved are complex: CKD and CVD share common risk factors, such as obesity, tobacco use and diabetes mellitus, while factors specific to CKD, such as anemia, abnormal pro-calcific milieu, uremia and chronic inflammation, can further aggravate CVD.

CVD is the leading cause of mortality globally, accounting for one-third of all global deaths [4], and with the added burden of CKD, mortality is 2- to 3-fold higher in CDV patients with abnormal renal function and/or proteinuria [3]. Overactivation of the renin–angiotensin system contributes to CVD by promoting inflammation, oxidative stress and endothelial dysfunction [5]. Therefore, adequate treatment is essential. Renin–angiotensin–aldosterone system (RAAS) inhibition with angiotensin-converting enzyme inhibitors (ACEi) or angiotensin receptor blockers (ARB) is a cornerstone of CVD treatment, especially in hypertension and heart failure. The use of ACEi reduces the risk of total mortality, cardiovascular mortality, non-fatal myocardial infarction and stroke [6], with ARBs exhibiting similar effects. Besides CVD benefits, ACEi and ARBs have demonstrated their benefits in CKD patients, lowering CKD progression and proteinuria despite the risk of hyperkalemia and the initial dip in the estimated glomerular filtration rate (eGFR) [7]. Conversely, in acute cases like sepsis, diarrhea or congestive heart failure, where there is a reduced adequate volume, the concomitant use of ACEi or ARB could lead to or aggravate acute kidney injury [8].

Over the past 20 years, angiotensin receptor neprilysin inhibitors (ARNI) have been developed, combining an ARB with a neprilysin inhibitor for added cardiovascular benefits. Neprilysin, a metalloprotease, degrades natriuretic peptides, particularly atrial natriuretic peptides. In heart failure, its increased activity accelerates peptide degradation, making neprilysin inhibition a crucial therapeutic target [5]. ARNI reduces major adverse cardiovascular event risk and outperforms ACEi and ARB, making it a key heart failure with reduced ejection fraction (HFrEF) treatment.

The renal effects of ARNI remain uncertain, highlighting the need for further investigation. With their growing use in other conditions, such as thrombotic microangiopathy, it is crucial to understand their renal outcomes and compare their benefits and drawbacks to traditional RAAS inhibitors. Our systematic review and meta-analysis aims to compare the renal effects of ARNI versus RASI.

## MATERIALS AND METHODS

In the present systematic review, we adhered to the revised guidelines provided by the Preferred Reporting Items for Systematic Reviews and Meta-Analyses (PRISMA) [9]. The PRISMA checklist is reported in Supplementary data, Table S1. This encompassed all aspects, spanning from the methodology of our search procedure to data collection and presentation. The systematic review protocol was registered in the PROSPERO register of systematic reviews (No: CRD42024621604).

### Data sources and search strategy

Potentially relevant studies were searched in the following databases from inception to 30 December 2024: MEDLINE (PubMed), Embase and Scopus. Language filters were not applied in the search process. In addition to the above sources, Google Scholar and ClinicalTrials.gov databases were screened for additional citations. References from representative studies were also researched to retrieve further studies for eligibility assessment. We used different combinations of keywords and controlled vocabulary to create a comprehensive search strategy: “sacubitril valsartan ACE,” “Sacubitril valsartan renin-angiotensin-aldosterone,” “neprilysin inhibitor,” “ARNI renal” and “ARNI RASI.”

### Eligibility criteria and outcomes

Two independent investigators decided whether to include eligible studies in the present systematic review and meta-analysis based on several prespecified inclusion and exclusion criteria. We established the following inclusion criteria before performing the search in the databases and data extraction: (i) studies with a randomized controlled design, (ii) studies that included humans ≥18 years old, (iii) patients were assigned to receive either a neprilysin inhibitor or RASI, with the latter used as the control group, and (iv) studies providing data regarding renal outcomes [rise in creatinine (Cr) levels, proteinuria, end-stage renal disease (ESRD), change in eGFR]. We included all eligible studies in the systematic review, regardless of whether they reported all outcomes of interest. Case reports, editorials, studies with overlapping populations, unpublished data and meta-analyses were excluded. Any possible disagreements were solved by discussion and consensus.

The primary outcomes included: ≥50% reduction in eGFR or ESRD, a composite of worsening renal function, defined as an increase in serum Cr of ≥0.5 mg/dL from baseline, and a decrease in eGFR of 25% from baseline and renal impairment, defined as an increase of at least 0.3 mg/dL in Cr values. Secondary outcomes included hyperkalemia (an increase of at least 5.5 mmol/L in serum potassium).

### Data collection and synthesis

After eligibility assessment and the inclusion of studies in the present systematic review and meta-analysis, two independent investigators extracted the following data: first author, publication year, study design, number of patients included in the neprilysin inhibitor group and the renin–angiotensin–aldosterone inhibitor group, age, investigated outcomes and number of events, and follow-up duration.

The pooled effect size, respectively odds ratio (OR), and corresponding 95% confidence intervals (CIs) were obtained by using Review Manager (RevMan) version 5.4.1 (Nordic Cochrane Centre, The Cochrane Collaboration, 2020, Copenhagen, Denmark). For this purpose, the random-effect model and Mantel–Haenszel method were used in dichotomous data. The heterogeneity of included studies was assessed by using I^2^ statistics, as follows: 0%–25% (low), 26%–50% (moderate), 51%–75% (high) and >75% (very high). A *P*-value lower than the threshold of 0.05 was considered to be significant.

### Quality assessment

The quality assessment of the studies included was conducted based on their design. Given that the included studies were clinical randomized trials, the risk of bias was evaluated using the revised Cochrane risk-of-bias tool for randomized trials (RoB 2) [11]. Additionally, publication bias will be assessed using funnel plot asymmetry and quantitatively evaluated using Egger's regression test. The quality of the evidence for each outcome was further assessed using the Grading of Recommendations Assessment, Development and Evaluation (GRADE) approach.

## RESULTS

**Figure 1:**
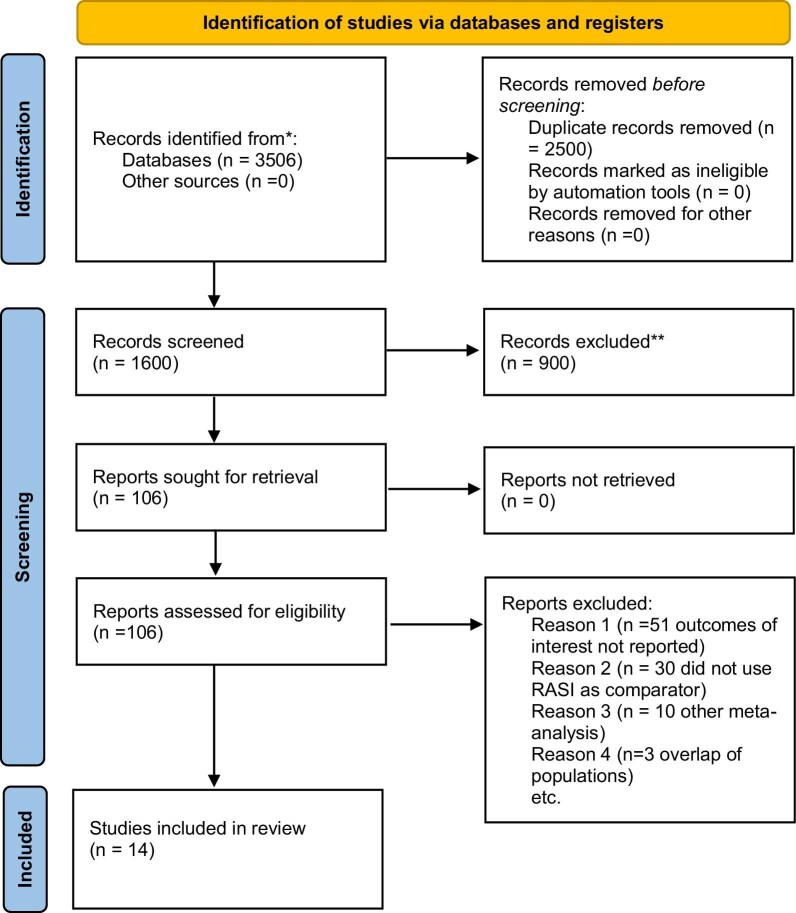
Flow diagram of selected studies in the present analysis.

A thorough and systematic search was conducted across designated databases, yielding an initial pool of 3506 records. Subsequently, duplicate publications were eliminated, resulting in a filtered dataset comprising 106 references. These articles underwent a second assessment in line with predefined inclusion and exclusion criteria based on a two-step approach. In the first step, two independent investigators evaluated titles and abstracts for eligibility criteria. The second step examined references that fulfilled the inclusion criteria in full text. After the full-text screening, 14 studies were included in the analysis [10–23]. The flowchart of the study can be seen in Fig. 1.

General data [number of participants randomized to ARNI or RASI, follow-up period, mean eGFR and urinary albumin-to-Cr ratio (UACR)] are reported in Table 1. Specific renal outcomes reported throughout studies are presented in Table 2. Regarding the population of the studies, eight studies included a population with heart failure and reduced ejection fraction (HFrEF) [11, 12, 14, 16, 17, 20, 22, 23], while four studies included a population with heart failure with preserved ejection fraction (HFpEF) [11, 13, 15, 21] and one study included a population with heart failure and preserved ejection fraction or moderately reduced ejection fraction [10]. Two studies did not have a population with heart failure [18, 19]. Regarding the type of RASI used, five studies used an ACEi [11, 12, 14, 22, 23], eight studies used an ARB [10, 13, 15–20] and one study used either ACEi or ARB [21]. In terms of which ARNI was used, only two studies used omapatrilat [22, 23], while the rest used sacubitril/valsartan.

**Table 1:** Baseline characteristics of included studies.

							Mean eGFR (mL/min/1.73 m^2^)		UACR (mg/mmoL)	
Study (author/year)	No. patients	ARNI	RASI	Type of RASI	HF	Follow-up	ARNI	RASI	ARNI	RASI
1. Damman *et al*. 2018	8442	4187	4212	ACEi	HFrEF	27M	ARNI + RASI		No CKD	CKD
							No CKD	CKD		
							81 ± 14	49 ± 8	1 (0.5–3.2)	1.6 (0.5–5.1)
2. Desai *et al.* 2019	464	232	233	ACEi	HFrEF	12W	70	69	NR	
3. Haynes *et al.* 2018	414	207	207	ARB	No HF	12M	35.4	35.5	ARNI	RASI
									52	56
4. Kang *et al.* 2019	118	60	58	ARB	HFrEF	12M	Serum Cr		NR	
							0.98 ± 0.28	1 ± 0.32		
5. Mann *et al.* 2021	335	167	168	ARB	HFrEF	24W	63.6	65.7	NR	
6. Mentz *et al.* 2023	466	233	233	ARB	HFm/pEF, worsening HF	20M	47.4 (Cr 1.3)	51.1 (Cr 1.2)	NR	
7. Packer *et al.* 2002	5770	2886	2884	ACEi	HFrEF	14.5M	NR	NR	NR	
8. Pieske *et al.* 2021	2572	1281	1285	ACEi or ARB placebo	HFpEF	24W	62.5	62.7	NR	
9. Rakugi *et al.* 2021	1161	ARNI 200 mg 387 ARNI 400 mg 385	389	ARB	No HF	8W	eGFR 30–60 ARNI 200 mg: 90 (23.3%)	eGFR 30–60: 131 (33.7%)	NR	
							eGFR 30–60 ARNI 400 mg: 113 (29.4%)			
							eGFR 60–90: ARNI 200 mg: 275 (71.1%)	eGFR 60–90: 242 (62.2%)		
							60–90 ARNI 400 mg: 250 (64.9%)			
							eGFR >90: 22 (5.7%) ARNI 200 mg	eGFR >90: 16 (4.1%)		
							eGFR >90: 22 (5.7%) ARNI 400 mg			
10. Rouleau *et al.* 2018	716	289	284	ACEi	HFrEF	10M	Serum Cr (µmol/L)		NR	
							103.4	101.7		
11. Solomon *et al.* 2012	266	134	132	ARB	HfpEF	12W	67	64	NR	
12. Solomon *et al.* 2019	4796	2407	2389	ARB	HfpEF	8M	63 ± 19	62 ± 19	NR	
13. Tsui Tsui *et al.* 2021	225	112	113	ACEi	HFrEF	33.9	58.3	57.6	NR	
14. Velasques *et al.* 2019	881	440	441	ARB	HFrEF	8W	58.4	58.9	NR	

M, months; W, weeks; NR, not reported; HF, heart failure.

**Table 2:** Renal outcomes throughout included studies.

Study	Renal outcomes	ARNI	RASI	Results
1. Damman *et al.* 2018	Prespecified composite renal outcome: 50% decline in eGFR relative to baseline, >30 mL/min/1.73 m^2^ decrease in eGFR to <60 mL/min/1.73 m^2^, ESRD	94/4187	108/4212	HR 0.86 (0.65–1.13), *P* = .29
	≥50% decrease in eGFR	32/4187	42/4212	HR 0.75 (0.48–1.19), *P* = .23
	>30 mL/min/1.73 m^2^ decrease in eGFR to <60 mL/min/1.73 m^2^	77/4187	69/4212	HR 1.11, *P* = .54
	ESRD	8/4187	16/4212	HR 0.50 (0.21–1.16), *P* = .11
	Post-hoc composite renal outcome: ≥50% reduction in eGFR or ESRD	37/4187	58/4212	HR 0.63 (0.42–0.95), *P* = .028
	UACR (1M) increase >25%	–1.35 (–1.82 to –0.88)	–2.37 (–2.89 to –1.85)	1.02 (0.32 to 1.72), *P* = .04
2. Desai *et al.* 2019	Worsening renal function: decrease in eGFR of ≥35% or increase in serum Cr of ≥0.5 mg/dL from baseline AND decrease in Egfr of ≥25% from baseline	12/231	14/233	RR 0.86 (0.41–1.83)
	Hyperkalemia	37/231 (16%)	30/233 (12.9%)	RR 1.24 (CI 0.80,1.94)
3. Haynes *et al.* 2018	Mean mGFR at 12 months	29.8 mL/min/1.73 m^2^	29.9 mL/min/1,73 m^2^	Difference in means (SE): –0.1 (*P* = 0.86)
	UACR	16.6 mg/mmol	17.9 mg/mmol	Difference in means: –9% (*P* = .08)
	≥25% reduction in CKD-EPI eGFR	71/207	67/207	*P* = .75
	Hyperkalemia	66/207 (32%)	50/207 (24%)	*P* = .10
4. Kang *et al*. 2019	Change in Cr levels at 12 months	–0.06 ± 0.61	0.09 ± 0.21	–0.03 (–0.30 to 0.97), *P* = .51
	Acute renal failure	1/58	0/60	*P* = .49
	Cr ≥2.5 mg/dL	2/60	2/58	*P* = 1
	Hyperkalemia	–0.06 ± 0.61	0.04 ± 0.42	Change: –0.10 (–0.30 to 0.97), *P* = .58
5. Mann *et al.* 2021	Worsening kidney function: eGFR <20 mL/min/1.73 m^2^ between randomization and week 24.	7/167	7/167	OR = 0.99 (0.34–2.91), *P* = .99
	Hyperkalemia	28/167 (17%)	15/168 (9%)	RR 2.05 (1.05–4), *P* = .04
6. Mentz *et al.* 2023	Worsening renal function composite: endpoint of renal death, ESRD or ≥50% decline in eGFR	50/233 (21.5%)	72/233 (30.9%)	OR 0.61 (0.40–0.93)
		34/27	46/35	RR 0.62 (0.25–1.56)
	Worsening renal function: Cr ≥0.5 mg/dL and ≥25% decrease in eGFR	50/233 (21.5%)	72/233 (30.9%)	OR 0.61 (0.40–0.93)
	Renal death	0	0	
	ESRD	20/20	22/22	
	≥50% decline in eGFR	14/14	22/22	
	Hyperkalemia	45/233 (19.3%)	43/233 (18.5%)	OR 1.06 (0.66–1.68)
7. Packer *et al.* 2002	Impaired renal function	196 (6.8%)	291 (10.1%)	

8. Pieske *et al.* 2021	25% decrease eGFR	26.2%	20.2%	25.7%	23.7%
	>40% decrease eGFR	6.6%	3.8%	6.2%	6%
	>50% decrease eGFR	2.3%	1%	2.5%	9%
	>30 mL/min/1.73 m^2^ decrease eGFR	5.3%	1.9%	5.3%	5.2%
	>60 mL/min/1.73 m^2^ decrease eGFR	0.6%	0%	0.2%	0.7%
	Cr ≥0.5 mg/dL	8.5%	6.2%	9.5%	9.1%
	Cr ≥2 mg/dL	7.8%	5.3%	9.5%	9.1%
	Hyperkalemia	20.1%	14.9%	19.3%	14.4%
9. Rakugi *et al.* 2021	Cr ≥2 mg/dL	0	0		
	Hyperkalemia	7/387 (1.8%)–200 mg 6/385 (1.6%)–400 mg	3/388 (0.8%)		
10. Rouleau *et al.* 2018	Elevated Cr (>1.5× baseline and 0.8× normal value)	5/289	17/284	The difference is not significant, *P* = .009	
	Hyperkalemia	6/289 (2.1%)	10/284 (3.6%)	Non-significant	
11. Solomon *et al.* 2012	Renal dysfunction	3/149	7/152	*P* = .34	
	eGFR decrease	–1.6 mL/min/1.73 m^2^	–5.2 mL/min/1.73 m^2^	*P* = .07	
	≥50% decrease in eGFR	5/149 (3%)	3/152 (3%)	*P* = .98	
	UACR	1.9 mg/mmol to 2.9 mg/mmol	2 mg/mmol baseline to 2 mg/mmol	*P* = .02	
	Hyperkalemia	24/140(16%)	16/152 (11%)	*P* = .21	
12. Solomon *et al.* 2019	Renal outcome composite: death from renal failure, ESRD, decrease of eGFR of 50% or more from baseline	33/2407	64/2389	HR 0.50 (0.33–0.77)	
	≥50% decrease in eGFR	33/2407	64/2389	HR 0.50 (0.33–0.77)	
	Renal impairment	301/2419 (12.44%)	356/2402 (14.82%)		
	Cr ≥2 mg/dL	261/2407 (10.8%)	328/2389 (13.7%)	*P* = .02	
	Cr ≥2.5 mg/dL	97/2407 (4%)	109/2389 (4.6%)	*P* = .36	
	Hyperkalemia	316/2386 (13.2%)	361/2367 (15.3%)	*P* = .048	
13. Tsui Tsui *et al.* 2021	Cr ≥2 mg/dL	7/111 (6.3%)	11/112 (9.8%)	*P* = .4619	
	Cr ≥2.5 mg/dL	2/111 (1.8%)	4/112 (3.6%)	*P* = .6832	
	Hyperkalemia	8/111 (7.2%)	6/112 (5.4%)	*P* = .5944	
14. Velasques *et al.* 2019	Worsening renal function: increase in the Cr ≥0.5 mg/dL and ≥25% decrease in eGFR	60/440	65/441	RR 0.93 (0.67–1.28)	
	Hyperkalemia	51/440 (11.6%)	41/441 (9.3%)	RR 1.25 (0.84–1.84)	

RR, risk ratio.

All studies included in the systematic review reported renal outcomes, with our meta-analysis focusing on those predefined in our search strategy. Renal outcomes were reported as follows: renal composite outcome [10, 12, 13, 14, 15, 17, 21], a composite of worsening renal function [10, 12, 14, 16, 17, 21] and ESRD [10, 14]. Other studies report mean mGFR [19], acute renal failure or impaired renal function [20, 22], elevated Cr levels [11, 18, 23] or changes in eGFR. Only three of the included studies had a CKD population: Damman *et al*. [14] had a subgroup of CKD population, Haynes *et al*. [19] included only CKD participants with a mean eGFR of 35.4–35.5 mL/min/1.73 m^2^ and Pieske *et al*. [21] included a subgroup of patients with CKD. It is important to mention that Pieske *et al*. compares ARNI with either ACEi, ARB or placebo.

Four studies [11, 13, 18, 21] reported Cr values >2 mg/dL as adverse events, and data suggested that no important differences were reported between ARNI and the use of ACEi or ARB. Three other studies [10, 14, 21] reported ≥50% decrease in eGFR with no clear evidence of a difference between ARNI and RASI.

Four studies [14, 15, 19, 21] assessed proteinuria using the UACR. However, a pooled analysis could not be conducted due to variations in how the data were reported across studies. In Haynes *et al*. [19], there was no apparent difference in UACR between treatment arms (study average difference –9%, 95% CI). In Damman *et al*. [14], an increase of ≥25% in the UACR at 1 and 8 months was more common in the ARNI group compared with RASI (46%, 51% versus 39%, 39%, *P* = .004 and *P* < .0001, respectively). However, in the same study, a 25% increase in UACR was associated with a higher risk of renal composite endpoint in the enalapril arm [hazard ratio (HR) 2.53, 95% CI 1.09–5.84] but not in the ARNI group (HR 0.28, 95% CI 0.08–1.01, *P* = .005). Solomon *et al*. [15] reported an increase of UACR in the ARNI group (1.9 mg/mmol at baseline to 2.9 mg/mmol at Week 36) compared with the RASI group (2 mg/mmol to 2 mg/mmol at Week 36, *P* = .02). Pieske *et al*. [21] reported a UACR increase in the Supplementary data, Table.

### Primary outcomes

#### A ≥50% reduction in eGFR or ESRD

**Figure 2:**
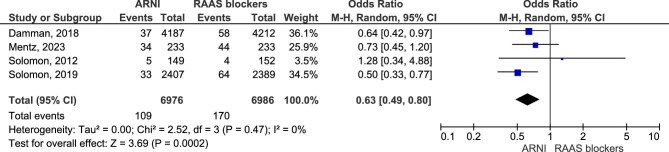
A≥50% decline in eGFR or ESRD.

Regarding this outcome, the analysis included four studies with a total number of 16 503 participants (6976 in the ARNI group and 6986 in the RAAS blockers group). Results showed that treatment with ARNI may lower the odds of ≥50% reduction in eGFR or ESRD (OR = 0.63, 95% CI 0.49–0.80, *P* = .0002) (Fig. 2).

#### Worsening renal function

**Figure 3:**
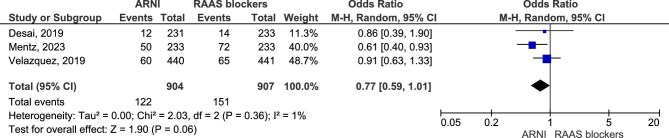
Worsening renal function.

Regarding the worsening renal function outcome (defined as an increase in serum Cr of ≥0.5 mg/dL from baseline and a decrease in eGFR of 25% from baseline), three studies were included in the pooled analysis, comprising a total of 1811 participants (904 in the ARNI group and 907 in the RAAS blockers group). Results showed no clear evidence that ARNI treatment affected the worsening renal function outcome (OR = 0.77, 95% CI 0.59–1.01, *P* = .06) (Fig. 3).

#### Renal impairment

**Figure 4:**
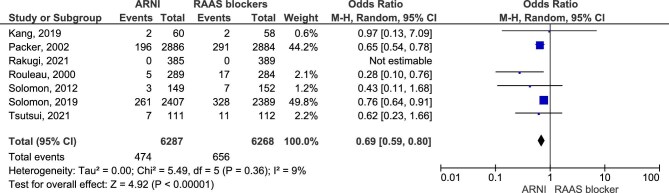
Renal impairment.

Seven studies were included in the analysis regarding one of the primary outcomes (renal impairment defined as an increase of at least 0.3 mg/dL in serum Cr). The results showed an advantage of ARNI compared with RASI regarding the odds of renal impairment (OR = 0.69, 95% CI 0.59–0.80, *P* < .00001) (Fig. 4).

**Figure 5:**
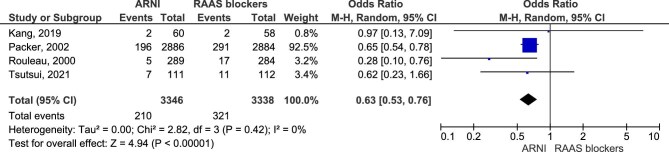
Subgroup analysis of renal impairment in HFrEF.

Next, a subgroup analysis that included studies with a population with heart failure with reduced ejection fraction was done, with results suggesting a lower odds of renal impairment in this population with ARNI treatment (OR = 0.63, 95% CI 0.53%–0.76, *P* < .00001) (Fig. 5).

### Secondary outcomes

#### Hyperkalemia

**Figure 6:**
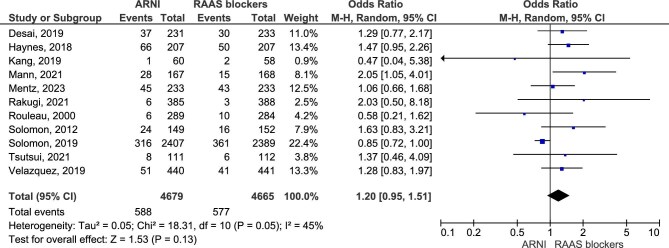
Hyperkalemia.

Regarding the secondary outcome of hyperkalemia, the analysis included 11 studies. The pooled OR was 1.20 (95% CI 0.95–1.51, *P* = 0.13), indicating that the data do not provide sufficient evidence to conclude a difference in the risk of hyperkalemia between ARNI and RASI treatment (Fig. 6).

### Risk of bias and publication bias

**Figure 7:**
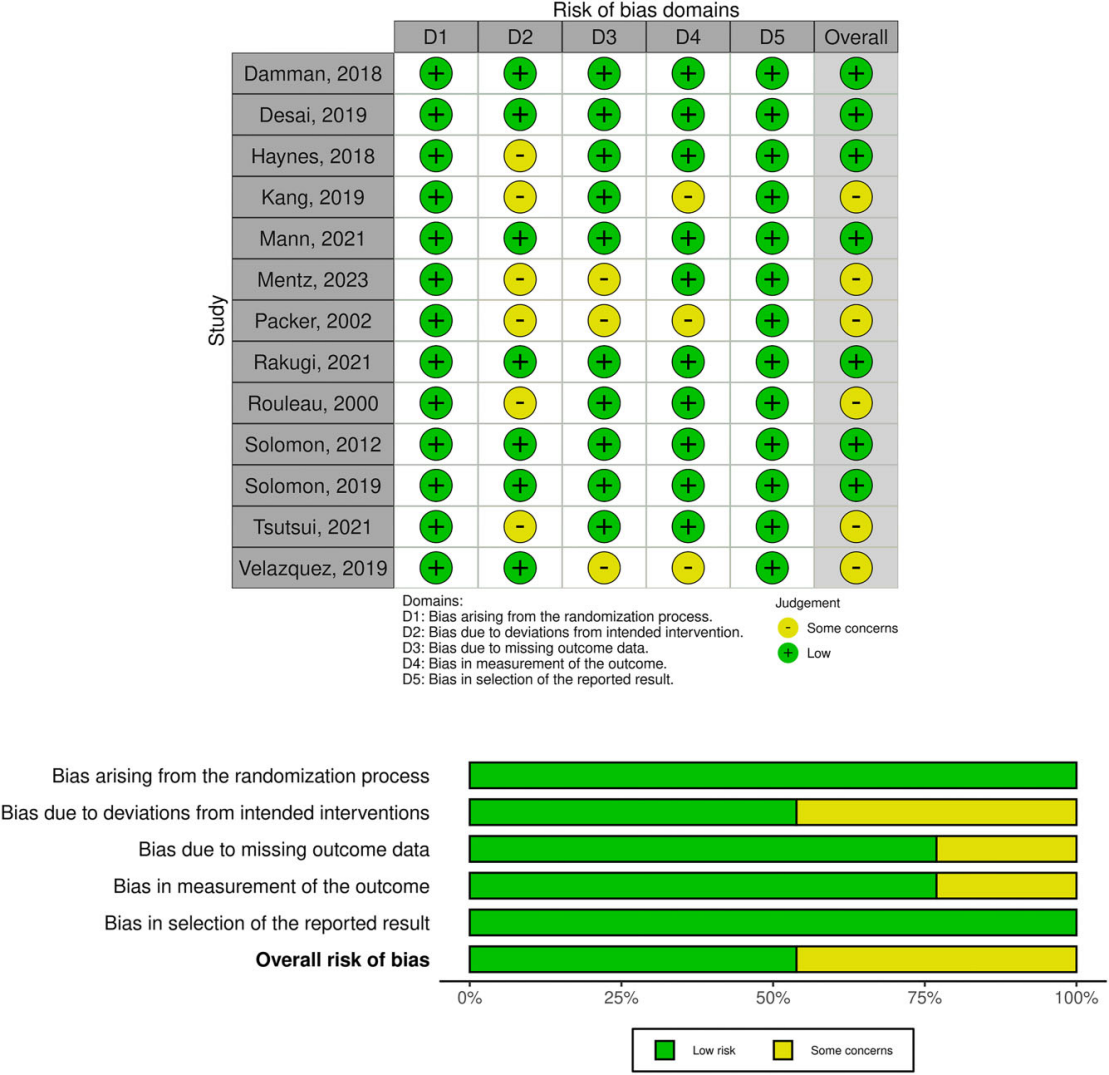
Risk of bias.

**Figure 8:**
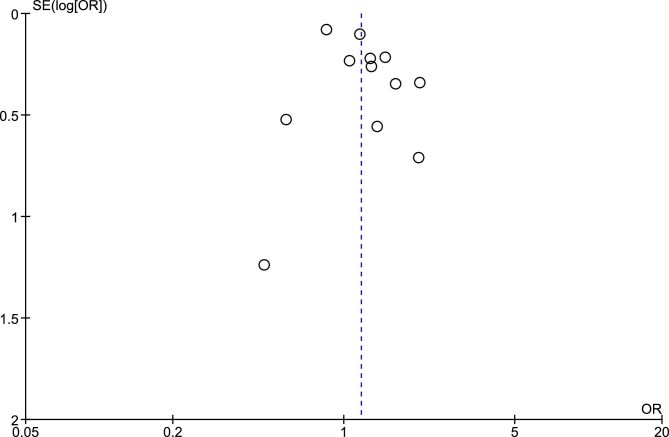
Funnel plot assessing publication bias.

The risk of bias was assessed using the RoB 2 tool (Figs 7 and 8). Publication bias was assessed for each outcome using Egger test. The results revealed no statistically significant funnel plot asymmetry for any of the outcomes analyzed: renal impairment (z = –0.99, *P* = .31), ≥50% reduction in eGFR or ESRD (z = 1.01, *P* = .31), worsening renal function (z = 0.23, *P* = .81) and hyperkalemia (z = 0.75, *P* = .45). Following these assessments, the overall quality of the evidence for each outcome was evaluated using the GRADE approach (Table 3). The findings of the GRADE assessment are summarized in Table 3, which highlights the certainty of evidence ranging from high to very low across the primary and secondary outcomes.

**Table 3:**
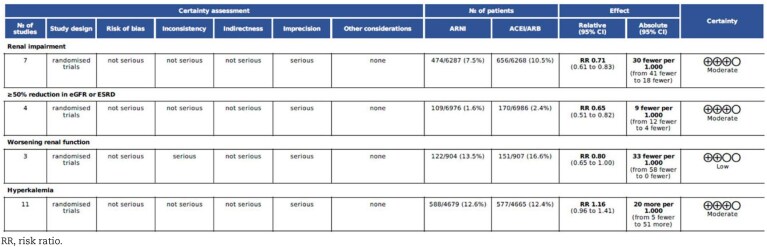
GRADE tool assessment.

## DISCUSSION

Our study found that treatment with ARNI was associated with a 37% lower odds of ≥50% reduction in eGFR or ESRD (OR = 0.63, 95% CI 0.49–0.80, *P* = .0002) and a 31% lower odds of renal impairment (OR = 0.69, 95% CI 0.59–0.80, *P* < .00001). Regarding the worsening renal function outcome, results showed no clear evidence that ARNI had an effect (OR = 0.77, 95% CI 0.59–1.01, *P* = .06). When considering a HFrEF population, our results showed a 37% lower odds of renal impairment that puts ARNI at an advantage (OR = 0.63, 95% CI 0.53%-0.76, *P* < .00001).

Regarding hyperkalemia, the data does not provide sufficient evidence to conclude a difference in the risk of hyperkalemia between ARNI and RASI treatment.

A previous meta-analysis published in 2020 evaluated the renal safety and efficacy of ARNI compared with RASI [24]. It included 11 randomized controlled trials, and results suggested that ARNI may offer advantages over RASI regarding renal dysfunction, eGFR decline and elevated serum potassium levels. However, despite the large sample size, the studies included did not clearly define renal dysfunction. Compared with our analysis, the metanalysis performed additional analysis regarding renal dysfunction (at >3 months and at <3 months). Additional studies are included in the pooled analysis based on specific renal outcomes. Another systematic review and meta-analysis published more recently [25] included 11 randomized controlled trials, evaluated renoprotection of ARNI versus RASI among participants with heart failure, and performed a subgroup analysis based on the type of heart failure (HFrEF and HFpEF). However, there was no clear definition of renoprotection in the pooled analysis. In the current study, we aimed to define renal outcomes clearly and analyzed the data available accordingly. Table 4 outlines the differences between previously published metanalysis and the current study.

**Table 4:** Differences between our metanalysis and the previous ones.

	No. RCT included	Outcomes	Results
Current systematic review and metanalysis	14	Renal impairment: at least 0.3 mg/dL increase in SCr	7 studies included (12 555 participants) OR 0.69 (0.59–0.80), *P* < .00001
		Worsening renal function: increase in serum Cr of ≥0.5 mg/dL from baseline and a decrease in eGFR of 25% from baseline	3 studies (1811 participants) OR 0.77 (0.59–1.01), *P* < .00001
		≥50% decrease in eGFR or ESRD	3 studies (13 962 participants) OR 0.63 (0.49–0.80), *P* = .0002)
		Hyperkalemia	11 studies (9334 participants) OR 1.2 (0.95–1.51), *P* = .13
Yu Feng *et al*. (2020)	11	Renal dysfunction: not defined	9 studies (21 716 participants) RR 0.72, 95% CI 0.64–0.82, *P* < .05 (favors ARNI)
		Decrease in eGFR: not defined.	3 studies (9114 participants) RR 0.08, 95% CI 0.03–0.12, *P* < .05 (favors ARNI)
		Elevated serum Cr: not defined	2 studies (13 195 participants) RR 0.82 (0.63–1.07)
		Hyperkalemia	8 studies (15 903 participants) RR 0.95, 95% CI 0.88 1.02, *P* > .05
		UACR	3 studies (2587) RR 0.32 (95% CI 0.12–0.51, *P* < .05) (favors ARNI)
Yang *et al*. (2024)	11	Renoprotective effects of ARNI: not defined	11 studies RR 0.86 (0.78–0.96), *P* = .016

RCT, randomized controlled trial; RR, risk ratio.

A strength of our meta-analysis is the inclusion of 14 randomized controlled trials, each reporting the renal outcomes we defined previously. However, there are some limitations. One limitation is considerable heterogeneity in reporting renal outcomes across studies, coupled with the absence of a universally accepted definition for adverse renal outcomes. There is a lack of consistency in the definition of acute kidney failure among studies, with some studies reporting rises in Cr levels >0.5 mg/dL from baseline and others reporting a rise in 0.3 mg/dL in Cr levels from baseline. Moreover, worsening kidney function is reported as increased Cr (0.3 mg/dL or 0.5 mg/dL), ESRD or eGFR <20 mL/min/1.73 m^2^. Regarding composite kidney outcome, studies reported a composite including a 50% decline in eGFR relative to baseline, > 30 mL/min/1.73 m^2^ or ESRD. These important differences limited the number of studies included in the pooled analysis. Another limitation is that most studies included in our systematic review and meta-analysis were primarily focused on patients with heart failure, as ARNI is a cornerstone therapy for heart failure with reduced ejection fraction. Lastly, we noted as a limitation that only one study specifically included CKD patients [19] and two studies included a subgroup of CKD population, but we could not provide a pooled analysis due to the data heterogeneity.

Recent data evaluated kidney outcomes following ARNI compared with ACEi/ARB in patients with thrombotic microangiopathy and malignant hypertension [26]. This cohort study evaluated a positive kidney outcome, defined as a composite of kidney recovery, including a 50% reduction in Cr levels, return to reference range Cr or kidney survival without dialysis for over 1 month. Results showed that ARNI treatment led to a quicker achievement of the primary outcome compared with ACEi and ARB. We considered this an important observation, given that the patients included in this study had acute kidney failure secondary to thrombotic microangiopathy and had a mean eGFR of 8.2 mL/min/1.73 m^2^ (ARNI group) and 12.2 mL/min/1.73 m^2^ (ACEi/ARB group).

Regarding ARNI use in CKD, a single study focused on the CKD population [19], where ARNI was compared with irbesartan. ARNI was similar to irbesartan, with an additional effect on lowering blood pressure and cardiac biomarkers. A recent meta-analysis evaluated the effect of ARNI in patients with heart failure and ESRD on maintenance dialysis [27], showing that ARNI treatment was well-tolerated, improved left ventricular function and reduced the risk of death for any cause with minimum adverse effects.

In recent years, ARNI has been explored for uses beyond heart failure, including post-myocardial infarction, arrhythmia and chemotherapy-induced left ventricular dysfunction, suggesting its effects may extend beyond the benefits seen in heart failure with reduced ejection fraction [28]. Therefore, additional studies are needed to fully elucidate the extent of the benefits associated with ARNI treatment. However, there is a lack of studies regarding the use of ARNI in a population with CKD or comparisons between ARNI treatment and the classical RAS inhibitors in patients with CKD with or without heart failure. A recent publication investigated the effect of ARNI on 24-h blood pressure in Japanese patients with non-dialysis advanced CKD with results suggesting that switching from ARB to ARNI can improve blood pressure control in patients with advanced CKD that do not achieve blood pressure goal [29].

In conclusion, our analysis showed that treatment with ARNI may reduce the odds of renal impairment by 31% and lowered the odds of ≥50% decrease in eGFR or ESRD by 37%, without increasing the risk of hyperkalemia when compared with RASI. Our findings did not demonstrate a clear effect on worsening renal function. In a subgroup of patients with HFrEF, ARNI was associated with a 37% lower odds of renal impairment compared with RASI. Further research is required to evaluate the renal effects of ARNI treatment, including its impact on kidney function, the risk of acute kidney injury, declines in eGFR and proteinuria. Additionally, there is a need for a universal definition of worsening kidney function and a standardized renal composite outcome. Additionally, studies focusing on a pure CKD population could provide further insight into the potential benefits of ARNI in this population.

## Supplementary Material

sfaf224_Supplemental_Files

## Data Availability

The data that supports the findings of this study are available on reasonable request from the corresponding author.
