# Supplementary material for: Comparative renal effects of angiotensin receptor neprilysin inhibitors and ACEi/ARB: a systematic review and meta-analysis
Source: Clin Kidney J. 2025 Jul 11;18(8):sfaf224. doi: 10.1093/ckj/sfaf224 (PMC12315105; doi:10.1093/ckj/sfaf224)
Supplement: sfaf224_Supplemental_Files [file sfaf224_supplemental_files.zip › Table S2. Databases and search strategy.docx]

| **Table S1.** Databases and search strategies used in present systematic review and meta-analysis. | | | | |
| --- | --- | --- | --- | --- |
| **Database** | **Coverage** | | **Search run** | **Records** |
| **MEDLINE** | 1946 to present | | neprilysin inhibitor AND ("Angiotensin-Converting Enzyme Inhibitors"[Mesh]) | 341 |
|  |  |  | (sacubitril valsartan OR neprilysin inhibitor OR ARNI) AND ("Angiotensin-Converting Enzyme Inhibitors"[Mesh] OR renin-angiotensin-aldosterone OR RASI) | 501 |
|  |  |  | sacubitril valsartan OR neprilysin inhibitor OR ARNI) AND ("Angiotensin-Converting Enzyme Inhibitors"[Mesh] OR renin-angiotensin-aldosterone OR RASI) AND (kidney OR renal) | 148 |
| Total records = 990 | | | | |
| **Embase** | 1966 to present | | neprilysin inhibitor AND (Angiotensin-Converting Enzyme Inhibitors) | 267 |
|  |  |  | (sacubitril valsartan OR neprilysin inhibitor OR ARNI) AND (Angiotensin-Converting Enzyme Inhibitors OR renin-angiotensin-aldosterone OR RASI) | 731 |
|  |  |  | (sacubitril valsartan OR neprilysin inhibitor OR ARNI) AND (Angiotensin-Converting Enzyme Inhibitors OR renin-angiotensin-aldosterone OR RASI) AND (kidney OR renal) | 262 |
| Total records = 1260 | | | | |
| **Cochrane library** | 1967 to present | | neprilysin inhibitor AND (Angiotensin-Converting Enzyme Inhibitors) | 82 |
|  |  |  | (sacubitril valsartan OR neprilysin inhibitor OR ARNI) AND (Angiotensin-Converting Enzyme Inhibitors OR renin-angiotensin-aldosterone OR RASI) | 143 |
|  |  |  | (sacubitril valsartan OR neprilysin inhibitor OR ARNI) AND (Angiotensin-Converting Enzyme Inhibitors OR renin-angiotensin-aldosterone OR RASI) AND (kidney OR renal) | 34 |
| Total records = 259 | | | | |
| **Scopus** | From the inception till present | neprilysin inhibitor AND (Angiotensin-Converting Enzyme Inhibitors) | | 535 |
|  |  | (sacubitril valsartan OR neprilysin inhibitor OR ARNI) AND (Angiotensin-Converting Enzyme Inhibitors OR renin-angiotensin-aldosterone OR RASI) | | 341 |
|  |  | (sacubitril valsartan OR neprilysin inhibitor OR ARNI) AND (Angiotensin-Converting Enzyme Inhibitors OR renin-angiotensin-aldosterone OR RASI) AND (kidney OR renal) | | 121 |
| Total records = 997 | | | | |
| All databases: 3662 records | | | | |
